# Professorships in child and adolescent psychiatry relative to a similarly sized medical specialty in the UK and Ireland: cross-sectional study

**DOI:** 10.1192/bjp.2024.179

**Published:** 2025-04

**Authors:** Ian Kelleher, Aleksandra Z. Poziemska, Valentina Kieseppä, Anita Thapar, Bernadka Dubicka, Elaine Lockhart, Tamsin Ford, Helen Minnis, Louise Gallagher, Fiona McNicholas, Kirstie O'Hare

**Affiliations:** Centre for Clinical Brain Sciences, Division of Psychiatry, University of Edinburgh, UK; School of Medicine, University College Dublin, Ireland; School of Medicine, University of Oulu, Finland; St John of God Hospitaller Services Group, Stillorgan, Ireland; Academic Child and Adolescent Psychiatry section and Wolfson Centre for Young People's Mental Health, Division of Psychological Medicine and Clinical Neurosciences, Cardiff University School of Medicine, UK; Department of Child and Adolescent Psychiatry, University of York, UK; Department of Psychiatry, Greater Manchester Mental Health NHS Foundation Trust, UK; Faculty of Biology, Medicine and Health, University of Manchester, UK; Faculty of Child and Adolescent Psychiatry, Royal College of Psychiatry, UK; Department of Psychiatry, University of Cambridge, UK; School of Health and Wellbeing, University of Glasgow, UK; Department of Psychiatry, The Hospital for Sick Children, Toronto, Canada; Centre for Addiction and Mental Health, Toronto, Canada; Department of Psychiatry, University of Toronto, Canada; Department of Psychiatry, Trinity College Dublin, Ireland; School of Clinical Medicine, University of New South Wales, Australia

**Keywords:** Academic psychiatry, child and adolescent psychiatry

## Abstract

**Background:**

A youth mental health crisis is considered one of the great challenges of our time, and research and clinical services in child and adolescent psychiatry have become a priority for governments and funders. Academic leadership is needed to drive forward research. It is not clear how many senior academic leadership posts (professorships) there are in child and adolescent psychiatry, nor how this benchmarks against a similarly sized medical specialty.

**Aims:**

This study aimed to determine the number of professorships in child and adolescent psychiatry in the UK and Ireland compared to a similarly sized specialty. A secondary aim was to identify the number of clinical trials registered for mental and behavioural disorders in children.

**Method:**

We identified registered specialists in child and adolescent psychiatry and a similarly sized specialty who held full professorships in medical schools. We searched the International Standard Randomised Controlled Trial Number (ISRCTN) and ClinicalTrials.gov for trials.

**Results:**

As of 23 March 2023, there were 1725 doctors on the General Medical Council's (GMC) specialist register in child and adolescent psychiatry. The closest specialty in terms of number of registered specialists was neurology (*N* = 1724). We identified 24 professors in child and adolescent psychiatry across the UK and Ireland, compared to 124 in neurology. For every intervention trial registered for mental and behavioural disorders in children, there were approximately ten trials registered for diseases of the nervous system.

**Conclusions:**

Despite equivalent numbers of medical specialists in child and adolescent psychiatry and neurology, there is a striking disparity in the number of professorship appointments. While young peoples’ mental health has, ostensibly, become a priority for policy-makers and funders, this is not reflected in medical professorship appointments. The paucity of senior academic child and adolescent psychiatrists has real-world implications for training, research, innovation and service development in mental health services.

The prevalence of mental disorders in young people has been rising in recent years, with exponential increases in referrals to specialist child and adolescent mental health services.^1,2^ This has frequently been referred to as a mental health crisis and is recognised as one of the great challenges of our time.^3^ There is an unprecedented focus on research and clinical services for children and young people's mental health from government, health and social care services and research funders.^4^ Healthcare service innovation and transformation are dependent on effective clinical research.^5,6^ With this in mind, we wished to determine the number of senior academic leadership posts (professorships) in child and adolescent psychiatry in medical schools in the UK and Ireland. Given recognition that there is an insufficient number of physician scientists across all areas of medicine,^7^ we also wished to compare the number of professorships in child and adolescent psychiatry to the equivalent number for a similarly sized specialty. As a related secondary aim, we also wished to identify the number of intervention trials being carried out for mental and behavioural disorders in children and adolescents compared to intervention trials for disorders related to the equivalent specialty.

## Method

This observational, cross-sectional analysis adhered to STrengthening the Reporting of OBservational studies in Epidemiology (STROBE) reporting guidelines.

In order to identify the number of doctors on the General Medical Council (GMC)'s specialist register, we used GMC ‘data explorer’ (an online tool available on the GMC's website: https://gde.gmc-uk.org/). We used this tool to identify the number of doctors on the specialist register for all medical specialties as of 23 March 2023. For the purposes of comparison, we chose the medical specialty with the closest number of doctors in the specialist register compared to child and adolescent psychiatry.

The GMC and Irish Medical Council (IMC) provided a list of accredited medical schools in the UK and Ireland, respectively. We searched the websites of each medical school between 23 March 2023 and 17 May 2023 and extracted information on professorships in child and adolescent psychiatry and the equivalent specialty. We subsequently contacted the deans of all medical schools to confirm or correct the details obtained in our online searches. In cases where we did not receive a reply, we sent a further follow-up email, and, if there was still no reply, we proceeded with the information on professorships that we had obtained from their official websites.

As we wished to identify the number of professorships, we searched only for information on full professorships (i.e. we did not include more junior academic posts). As we were interested in substantive university posts, we did not include emerita/emeritus, honorary or visiting professorships. As we were interested in senior academic leadership roles among physicians, we cross-checked the list of professors with the GMC or IMC's register of medical specialists for the UK and Ireland respectively, to confirm that the professor was a registered specialist in child and adolescent psychiatry or the equivalent specialty.

As a secondary outcome, we wished to investigate the number of intervention trials being carried out for mental and behavioural disorders in children and adolescents compared to intervention trials for disorders related to the equivalent specialty. To do this, we analysed data using the two clinical trials registers that are approved for use by the National Health Service (NHS) Health Research Authority: ClinicalTrials.gov and the International Standard Randomised Controlled Trial Number (ISRCTN; see https://www.isrctn.com/).

For ClinicalTrials.gov, we used the following search: terms: mental and behavioral disorders; study status: looking for participants; age range: child (0–17 years); location: United Kingdom; study type: interventional. We repeated the same search using Ireland as the location. For ISRCTN, we used the following search: term: mental and behavioural disorders; study status: ongoing; age range: child; recruitment country: United Kingdom. We repeated the same search using Ireland as the recruitment country. We adapted these search strategies for the comparison medical specialty (see the ‘Results’ section).

Descriptive statistical analyses were conducted using Microsoft Excel 2016 for Windows.

### Ethics statement

This study used publicly available data and was exempt from institutional review board review.

## Results

On 23 March 2023, there were 1725 doctors on the GMC's specialist register for child and adolescent psychiatry. The closest specialty in terms of number of doctors on the specialist register was neurology (*N* = 1724). This did not include paediatric neurology, which is recognised on the GMC register as a separate specialty.

The GMC provided a list of 37 medical schools which it accredits in the UK. The IMC provided a list of six medical schools which it accredits in Ireland. We extracted information on professorship appointments listed on each of the medical school websites. We then emailed the deans of all medical schools with this information and asked them to correct any inaccuracies in the data we had extracted. We received email responses from 26 (60.5%) of the medical schools. Where we did not receive a response, we progressed with the data that we had extracted from medical school websites.

In total, we identified 24 professorships in child and adolescent psychiatry and 124 professorships in neurology in medical schools in the UK and Ireland (see Table 1).

**Table 1:** Number of professorships in child and adolescent psychiatry and neurology in Ireland and the UK (with breakdown for the four nations of the UK)

	Professors in neurology	Professors in child and adolescent psychiatry
Ireland	9	2
England	102	17
Scotland	9	4
Wales	3	1
Northern Ireland	1	0
Total UK	115	22
Total UK and Ireland	124	24

Of the child and adolescent psychiatry professorships, 9 (37.5%) were held by women and 15 (62.5%) by men. For neurology, 18 professorships (14.5%) were held by women, 103 (83.0%) by men, and for 3 (2.4%) this information was unavailable.

### Intervention Trials

#### ClinicalTrials.gov

As of 31 January 2024, for mental and behavioural disorders in children, there were 30 active trials registered in the UK and 4 in Ireland. The comparison was diseases of the nervous system in adults aged 18 to 64 years (i.e. we did not include paediatric neurology, which is recognised by the GMC as a separate specialty, and we did not include those aged >65 years, which would include geriatric medicine). There were 426 active trials registered in the UK and 43 in Ireland.

#### ISRCTN

As of 31 January 2024, for mental and behavioural disorders in children, there were 18 active trials registered in the UK and 1 in Ireland. By comparison, for the category, diseases of the nervous system in adults (i.e. excluding trials in children and in individuals aged >65 years), there were 74 trials registered in the UK and 4 in Ireland.

## Discussion

We identified 1725 doctors on the GMC specialist register for child and adolescent psychiatry and 1724 for neurology. Despite an equivalent number of medical specialists, there was a major disparity in the number of senior academic leadership posts (professorships) across the two specialties, with five professorships in neurology for every one professorship in child and adolescent psychiatry.

The paucity of professorships in child and adolescent psychiatry has serious real-world implications for training, research and development of services and treatments for children's and adolescents’ mental health. Notably, we found a large difference in the number of intervention trials registered across the two areas: for every intervention trial registered for mental and behavioural disorders in children, there were approximately ten trials registered for diseases of the nervous system. Clinical research in the UK has been shown to drive healthcare service innovation, reduce healthcare inequalities and produce cutting-edge treatments.^6^ In this time of mental health crisis among young people, healthcare policy-makers and research funders hope to emulate this innovation for children and young people's mental health by making youth mental health a priority area, but this will not be possible without sufficient senior clinical academics to lead the necessary research.

Higher levels of research activity in clinical services are associated with a range of improved outcomes, including better healthcare service performance,^5^ greater patient confidence in clinical staff,^8^ better-informed patients,^8^ reduced patient mortality^9^ and improved doctor retention,^10^ something that is particularly important in the context of high levels of burnout among child and adolescent psychiatrists.^11^

It is important to note that research has fuelled major advancements in our understanding of neurobiology, the developing brain and the role of genetic and environmental factors in shaping mental health outcomes.^12^ Rapid technological advancements in the areas of clinical informatics, artificial intelligence and digital technologies have the potential to drive innovations and transformation of child and adolescent mental healthcare, but this requires urgent investment in research – and in research leadership posts.

Several factors may have contributed to the lower number of professorships in child and adolescent psychiatry compared to neurology, including the length and nature of training, different academic expectations for career progression, medical school priorities being biased towards physical health and, most importantly, funding. A recent review of research funding in Canada, for example, found that the research investment per year lived with disability for neurological disorders was C$571 compared with C$102 for mental health disorders (note this was for all mental health research, and not limited to child and adolescent research).^13^ The fact that neurology has been established as a specialty for significantly longer than child and adolescent psychiatry^14,15^ might also be considered to have played a role, but it is important to note that research in other relatively modern specialties, such as oncology, has thrived in line with heavy research investment, demonstrating that the issue is not explained by the age of the specialty.

## Limitations

Our information on professorships relied on information from medical school websites, which may not be fully up to date across all medical schools. We have no reason to believe, however, that under-recording of professorships on medical school websites would disproportionately affect one specialty over the other. In addition, we received feedback on the accuracy of our data from 60.5% of medical schools. Irrespective, any bias is likely to be towards underreporting the scale of the difference as – unsurprisingly, given the small pool – all professors in child and adolescent psychiatry in the UK and Ireland were known to several of this report's authors. On the other hand, it is possible that some professors in neurology could have been missed in the medical schools if their websites were not up to date and they did not respond to our request for information. The two trial registers used in the current study, ClinicalTrials.gov and ISRCTN, do not necessarily capture all healthcare intervention research taking place in the UK and Ireland. However, these are the two trial registers that are approved for use by the NHS Health Research Authority and so they are likely to capture intervention trials being conducted in NHS services across the UK.

### Future directions

Addressing the striking shortage of academic child and adolescent psychiatrists will require a major concerted and coordinated effort from stakeholders across government, academia, clinical service and research funding bodies. This means strategic, big-picture thinking that recognises that long-term solutions to a youth mental health crisis will not simply emerge from expanding existing clinical services but, rather, from research. This includes the need to build appropriate research infrastructures for child and adolescent psychiatry within universities, more long-term and sustained funding for the field and the creation of research opportunities across all career stages.

## Conclusion

There is a major disparity between health policy priorities and actual investment in senior academic leadership posts (professorships) within academic child and adolescent psychiatry in medical schools in the UK and Ireland. The lack of parity between physical and mental health is also underscored by the finding that there was only a fraction of the number of intervention trials registered for mental and behavioural disorders of childhood compared to diseases of the nervous system. In the context of a rising prevalence of mental health disorders in children and young people, urgent action is needed to address the lack of professorships in child and adolescent psychiatry.

## Data Availability

To access data from this study, please contact the corresponding author, I.K.
